# Determining the Exposure Pathway and Impacts of *Microcystis* on Threadfin Shad, *Dorosoma petenense*, in San Francisco Estuary

**DOI:** 10.1002/etc.4659

**Published:** 2020-02-21

**Authors:** Shawn Acuña, Dolores Baxa, Peggy Lehman, Foo‐Ching Teh, Dong‐Fang Deng, Swee Teh

**Affiliations:** ^1^ Metropolitan Water District of Southern California Sacramento California USA; ^2^ University of California, Davis Davis California USA; ^3^ California Department of Water Resources West Sacramento California USA; ^4^ University of Wisconsin, Milwaukee Milwaukee Wisconsin USA

**Keywords:** Histopathology, Algal toxins, Fish indices, Biomarkers

## Abstract

Blooms of the cyanobacterium *Microcystis* spp. could affect fish health through the ingestion of colonies as well as exposure to dissolved microcystins in the water column. The goal of the present study was to evaluate the dietary exposure pathway through which *Microcystis* spp. blooms may affect liver function and nutritional status using a novel approach involving multiple analytical methods to assess the potential risk. Our study was conducted using threadfin shad, *Dorosoma petenense*, which is a pelagic fish commonly exposed to *Microcystis* spp. blooms in the upper San Francisco Estuary. The approach incorporated published and optimized methods that offer multiple lines of evidence including in situ hybridization, immunohistochemistry, histopathology, condition factor indices, and nutritional profiles. Measurements of threadfin shad health and tissue condition were conducted at sites where *Microcystis* was present or absent during the 2007 bloom season. The results showed that dietary exposure to fish from *Microcystis* blooms resulted in the accumulation of microcystin in the gut and liver tissues of threadfin shad collected from the sites with blooms. Although toxicity endpoints were likely confounded by antecedent conditions, our findings demonstrate dietary exposure of *Microcystis* toxins to fish using a novel approach with multiple lines of evidence. *Environ Toxicol Chem* 2020;39:787–798. © 2020 The Authors. *Environmental Toxicology and Chemistry* published by Wiley Periodicals, Inc. on behalf of SETAC.

## Graphical abstract

Using a novel approach, the dietary exposure of microcystin to threadfin shad, *Dorosoma petenense*, during the presence of the *Microcystis* spp. bloom in the upper San Francisco Estuary was validated using multiple lines of evidence, and thus there is a risk of toxicity through ingestion of *Microcystis* spp. CVP = Central Valley Project.

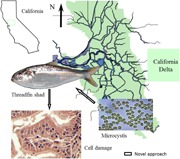

## INTRODUCTION

*Microcystis* spp. (*Microcystis*) is a cyanobacterium that can form cyanobacterial harmful algal blooms and dominate phytoplankton communities in fresh and brackish eutrophic waters such as rivers, estuaries, and lakes (Verspagen et al. 2006; Backer et al. 2008; Paerl and Huisman 2009). Blooms can produce natural toxins that promote liver tumors, neural toxicity, and developmental toxicity (International Agency for Research on Cancer 2006; Downing et al. 2011; Zegura et al. 2011; Zanchett and Oliveira‐Filho 2013). These natural toxins include lipopolysaccharides, neurotoxin β‐methylamino‐L‐alanine (Cox et al. 2005; Downing et al. 2011; Esterhuizen‐Londt et al. 2018), and the metabolite, microcystin. Microcystins have been found to cause impaired cellular function, lipidosis, sinusoidal congestion, and/or necrosis in the liver of common carp, *Cyprinus carpio* (Drobac et al. 2016), rainbow trout, *Oncorhynchus mykiss* (Tencalla et al. 1994), threadfin shad, *Dorosoma petenense*, (Acuña et al. 2012b) and medaka fish, *Oryzias latipes* (Mezhoud et al. 2008; Deng et al. 2010). Microcystins were also found to cause necrosis in the kidney, gill, gut, and muscle of common carp, *C. carpio* (Carbis et al. 1996; Drobac et al. 2016). Chronic exposure to microsystins has been shown to cause neurological and reproductive impairment and increased mortality in fish (Chen et al. 2016; Hu et al. 2016).

In the upper San Francisco Estuary (CA, USA), *Microcystis* commonly forms cyanobacterial harmful algal blooms accounting for up to 94.4% (v/v) of the bloom composition (Kurobe et al. 2018). Blooms in the San Francisco Estuary occur during the warm summer months of the year in fresh to brackish water bodies at salinities from 0.1 to 18 (Lehman et al. 2005; Kurobe et al. 2018). Furthermore, relatively high levels of microsystins (0.007–10.81 μg/L) occur in cyanobacteria during blooms (Baxa et al. 2010) and in fish prey (Lehman et al. 2005, 2013). Microcystins affect the mortality and species composition of zooplankton at the base of the food web (Ger et al. 2009a, 2009b, 2018). Laboratory bioassays have also demonstrated that Sacramento splittail, *Pogonichthys macrolepidotus*, and threadfin shad exposed to dietary *Microcystis* had impaired nutritional status and reduced condition factors, as well as elevated numbers of necrotic and deformed cells in liver tissue (Acuña et al. 2012a, 2012b). Furthermore, inland silversides (*Menidia beryllina*) and juvenile striped bass (*Morone saxatilis*) caught during *Microcystis* blooms demonstrated tissue lesions that were consistent with the presence of liver toxins such as microsystins (Lehman et al. 2010). Several studies have hypothesized that exposure to *Microcystis* and its associated microsystins has contributed to the decline in the abundance of native, endangered, and threatened fish species in the San Francisco Estuary after 2000, which coincided with the onset of *Microcystis* blooms (Armor et al. 2005; Malbrouck and Kestemont 2006; Sommer et al. 2007). However, the exposure pathways are unknown. Fish exposure to microsystins occurs through direct ingestion of the cyanobacteria (Tencalla et al. 1994), from their prey (De Magalthaes et al. 2001; Van der Oost et al. 2003), and from ambient exposure to environmental waters (Cazenave et al. 2005; Xie et al. 2005).

The purpose of the present study was to assess the potential exposure pathways through which *Microcystis* blooms may affect the liver function and nutritional status of threadfin shad in the San Francisco Estuary. We hypothesized that *Microcystis* toxicity occurs through the direct consumption of cyanobacterial colonies. The rationale for using the pelagic threadfin shad to test this hypothesis is that these fish are indiscriminate pelagic filter and particulate feeders (Holanov and Tash 1978) and therefore have the potential to feed directly on the colonies of cyanobacteria and/or on the grazers of the colonies such as zooplankton. The threadfin shad are present throughout the summer bloom period where they may consume *Microcystis*. Multiple exposure pathways to *Microcystis* and microsystins are likely to occur including filter feeding on small zooplankton and detritus particles, direct consumption of colonies, and exposure to dissolved microsystins during bloom senescence. In addition, *Microcystis* exhibits a diel vertical migration, which exposes threadfin shad to cyanobacteria that may occur throughout the water column. Using wild threadfin shad in the present study allowed us to use laboratory bioassays optimized for *Microcystis* and threadfin shad (Acuña et al. 2012b).

For the present study, *Microcystis* toxicity was assessed in threadfin shad via a dietary exposure pathway using methods that offer multiple lines of evidence: 1) in situ hybridization to determine the presence of *Microcystis* DNA in the intestine and gut contents via ingestion; 2) immunohistochemistry to determine the presence of microsystins in intestinal and liver tissues via digestion; 3) histopathology to determine tissue degeneration due to microsystin toxicity; and 4) nutritional composition analysis (RNA‐to‐DNA ratio and proximate composition) to assess the nutritional profiles. Integration of these methods that were previously developed and optimized in the laboratory would support field assessments of the dietary exposure pathways of fish to *Microcystis* toxicity and would aid in developing strategies for fish management during cyanobacterial harmful algal blooms. In addition, the results would help to inform the potential for future impacts of *Microcystis* blooms that are expected to increase with frequency and intensity due to climate change in the San Francisco Estuary, and worldwide (Intergovernmental Panel on Climate Change 2014).

## MATERIALS AND METHODS

### Description of fish collection area

**Figure 1:**
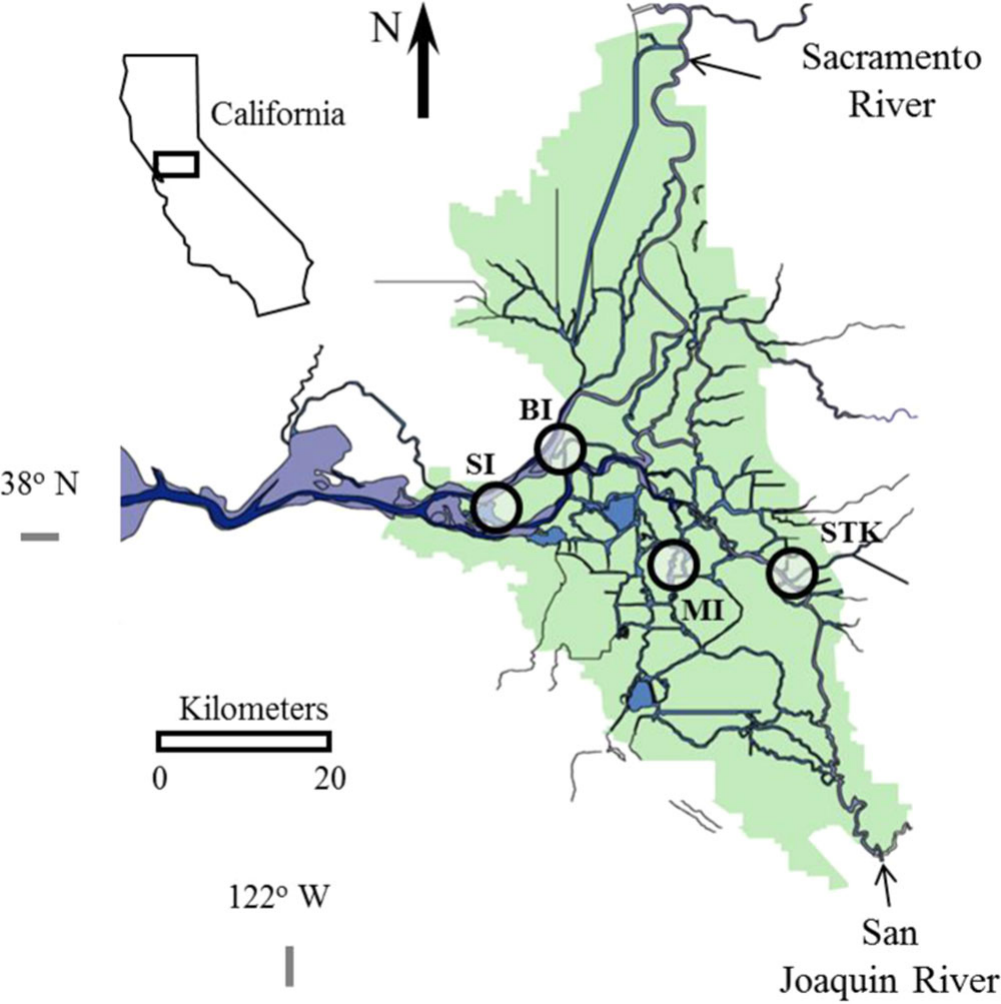
The California Delta showing the locations (circles) Sherman Island (SI), Brannon Island (BI), Mildred Island (MI), and Stockton (STK) for sampling threadfin shad, *Dorosoma petenense*.

The 4 sample collection sites are in areas that overlap threadfin shad and *Microcystis* distribution (Feyrer et al. 2009) including Sherman Island, Brannon Island, Mildred Island, and Stockton in the upper San Francisco Estuary (Figure 1). Sites Sherman Island and Brannon Island are both located at boat ramps within the Lower Sherman Island Wildlife Area and Brannon Island State Recreation Area, respectively. The Stockton site is located at a boat ramp in Louis Park adjacent to a highly urbanized and industrialized section of the San Joaquin River. The Mildred Island site is in the upper San Francisco Estuary within a submerged island that was flooded in 1983 and consists of open water with remnant riprap levees.

The upper San Francisco Estuary is a eutrophic watershed in the Central Valley of California. Ecological alteration of the upper San Francisco Estuary began in the mid‐nineteenth century with hydraulic mining, channelization, wetland reclamation, fish introduction, and water diversions (Sommer et al. 2007), and the Estuary currently consists of floodplains, submerged islands, deep channelized rivers, and tidal marshes. The upper San Francisco Estuary is mostly rural, with a majority of the population centered in the cities of Sacramento, West Sacramento, and Stockton and is primarily used for agriculture and wildlife habitat. *Microcystis* blooms occur throughout the upper San Francisco Estuary during the warmer months of the year (Boyer 2007; Lehman et al. 2013).

### Fish collection

Groups of threadfin shad were caught by beach seining from the 4 locations (Sherman Island, Brannon Island, Mildred Island, and Stockton) in the San Francisco Estuary between 28 August and 12 September 2007 during the peak of the *Microcystis* blooms (Figure 1 and Table 1). All fish were euthanized on site with an overdose of buffered tricaine methane sulfonate (Sigma). The euthanized fish were blotted dry with a paper towel for 1) necropsy; 2) freezing in dry ice, transportation to the University of California, Davis, and then storage at −80 °C until processing for proximate analysis; or 3) placement in 10% (v/v) neutrally buffered formalin for histopathology. Due to the unequal sample size between stations, priority was given to preservation for necropsies and formalin because both associated assays had been correlated with exposure in the laboratory study on threadfin shad (Acuña et al. 2012b).

**Table 1:** Number of threadfin shad, *Dorosoma petenense*, collected from the upper San Francisco Estuary for morphometric, histopathologic, and proximate analysis, and for determination of the RNA‐to‐DNA ratio^a^

Location	Total	Morphometrics	Histopathology	Proximate analysis
Sherman Island	42	20	15	7
Stockton	27	8	15	4
Brannon Island	34	15	15	4
Mildred Island	24	8	15	1^b^

^a^ Fish were collected from Sherman Island on 8/28/07 and 9/11/07, from Stockton on 8/29/07, from Brannon Island on 9/11/07, and from Mildred Island on 9/12/07.

^b^ Insufficient amount of sample to perform replicates.

Necropsied fish were observed for external condition (lesions, hemorrhaging, and deformities) and measured for liver weight (*W*_l_), body weight (*W*_b_), and fork length. Hepatosomatic index (HSI) and condition factor were used to determine the relative health of threadfin shad using the equation: HSI = 100 × *W*_l_/*W*_b_ and condition factor = 100 × *W*_b_/fork length^3^, respectively, where *W*_l_ and *W*_b_ is in grams, and fork length is in centimeters. Muscle tissue was removed from up to 10 threadfin shad from each site and placed in 90% ethanol (v/v) for RNA/DNA analysis.

### Microcystis presence/absence

The presence or absence of large *Microcystis* colonies at the surface of each sampling station during fish collection was determined by a visual abundance index that ranged from low to high (0–5), with 0 indicating no colonies and 5 indicating the presence of a thick algal scum. The Interagency Ecological Study Program (2019) has developed a qualitative index scoring method for estimating bloom severity for discrete water quality monitoring and summer tow net surveys. The qualitative scoring allowed for a rapid turnaround in the compliance monitoring data. The purpose of the compliance monitoring was for rapid assessment of lower trophic level dynamics that obviated more labor‐intensive methods such as cell counts or flow cytometry. Morphometric and genetic compositions of bloom colonies have been analyzed and determined to be predominantly *Microcystis* (Baxa et al. 2010; Lehman et al. 2013), which remains the most dominant cyanobacterial species to date in the San Francisco Estuary (Kurobe et al. 2018). Colonies contain a suite of microsystin congeners, but are often dominated by microsystin‐LR (Lehman et al. 2008).

Assessments of *Microcystis* presence were conducted at the sites during threadfin shad collections in August and September (Table 1). Colonies of *Microcystis* were collected from surface tows using a 0.5 m‐diameter plankton net fitted with a 75‐µm mesh net, for microsystin concentration analysis in the laboratory (Lehman et al. 2013). A wide mesh net was necessary due to high sediment load.

### Determination of microcystin concentration

Total microsystin concentration was determined from collected *Microcystis* colonies using liquid chromatography coupled with photodiode array and mass selective detection as described in Harada et al. (1999). *Microcystis* colonies were filtered with Millipore glass fiber filters GF/F from surface waters sampled across the sites. The filters were immediately frozen on dry ice and stored at –80 °C until processing for microsystin measurement at the University of California, Davis Puschner Laboratory.

### Histopathology

After 48 h in 10% (v/v) neutral buffered formalin, tissues were dehydrated in a graded ethanol series and embedded in paraffin. For each tissue block, serial sections (2–3 μm thick) were cut and stained with hematoxylin and eosin. Tissues were screened for various histopathological features and lesions including glycogen depletion, macrophage aggregate, lipidosis, eosinophilic droplets, infiltration of inflammatory cells, cytoplasmic inclusion bodies, sinusoidal congestion, and single‐cell necrosis according to the methods given in Teh et al. (2004). The lesions were scored on an ordinal ranking system of 0 = none to minimal, 1 = mild, 2 = moderate, and 3 = severe using a BH‐2 Olympus microscope. Glycogen depletion was characterized by decreased size of hepatocytes, loss of the “lacy,” irregular, and poorly demarcated cytoplasmic vacuolation typical of glycogen, and increased cytoplasmic basophilia (i.e., blue coloration). Fatty vacuolar degeneration or lipidosis was characterized by excess lipid that appeared as clear, round, and well‐demarcated cytoplasmic vacuoles. Macrophage aggregate was described as a cluster of macrophages packed with coarsely granular yellow–brown pigment. Eosinophilic droplets were characterized by the presence of proteins appearing as eosinophilic (pink coloration), round, and well‐demarcated cytoplasmic droplets. Infiltration of inflammatory cells was identified by focal‐to‐multifocal aggregates of lymphocytes that had infiltrated the connective tissue around bile ducts or blood vessels or parenchyma. Cytoplasmic inclusion bodies were characterized as unknown materials in the cytoplasm of hepatocytes. Sinusoidal congestion was identified as blood cell profusion in the interstitial spaces of the liver. Single‐cell necrosis in liver and germ cell necrosis in the testis and ovary were identified by the presence of cells (hepatocytes, spermatocytes, and oocytes) having eosinophilic (i.e., pink coloration) cytoplasm with nuclear pyknosis and karyorrhexis.

### In situ hybridization

Primers for *Microcystis* spp., MIC16S F (AAA GCG TGC TAC TGG GCT GTA) and MIC16S R (CCC TTT CGC TCC CCT AGC T), designed from the 16S rDNA sequences of *Microcystis* present in the San Francisco Estuary (Baxa et al. 2010) were used in the present study. Using these primer sets, the plasmid containing the partial *Microcystis* DNA polymerase was employed as a template (~50 pg) in the polymerase chain reaction (PCR) for generating the probe using conditions previously described (Baxa et al. 2010). The PCR assay for generating the *Microcystis* probe for the in situ hybridization (ISH) protocol (Baxa et al. 2010) included labeling with digoxigenin using a PCR digoxigenin probe synthesis kit (Roche Applied Science) according to the manufacturer's instructions. Following PCR, the labeled probe was gel‐confirmed for successful tagging with digoxigenin. Prior to use in the ISH assay, the probes were denatured for 5 min at 95 °C, immediately placed on ice, and stored at –20 °C until used. Except for modifications on the hybridization temperature (52 °C) and signal development at 4 °C overnight with the substrate solution, all steps in the ISH assay followed the method of Antonio et al. (1998). The PCR assay that was used to develop the ISH protocol for *Microcystis aeruginosa* has been previously validated and published (Baxa et al. 2010) and used for the assessment of threadfin shad exposed to *Microcystis* collected from the San Francisco Estuary (Acuña et al. 2012b). In the present study, histological sections of threadfin shad positive and negative for *Microcystis* were stained with labeled and unlabeled probes to validate the ISH assay. For detection and localization of *Microcystis* DNA by ISH, whole fish sections were processed to paraffin wax blocks, cut into 5‐μm‐thick sections, and then placed on Superfrost plus slides (Fisher Scientific).

### Nutritional analysis of threadfin shad

Proximate composition of the whole fish was used to indicate fish nutritional status in response to *Microcystis* exposure using methods described by the Association of Official Analytical Chemists (1995). For proximate composition, whole fish samples were freeze dried and homogenized using liquid nitrogen Freezer/Mill (SPEX SamplePrep). Percentages of moisture, protein, and lipid content were determined for each of the samples. Protein by total nitrogen was determined by the Kjeldahl method, and the crude protein was calculated by multiplying the value of the total nitrogen by 6.25. The lipid content was determined using the Soxhlet method.

Detoxification and liver damage from exposure to microsystin from *Microcystis* can be nutritionally stressful and can impair fish growth due to a lack of energy (Leao et al. 2009; Deng et al. 2010; Acuña et al. 2012b). Therefore, we used an RNA‐to‐DNA ratio of fish muscle tissue as an index of nutritional stress and growth (Caldarone et al. 2001). White muscle tissue from individual frozen fish were removed and measured for nucleic acid contents, RNA, and DNA, using the ethidium bromide fluorometric method (Caldarone et al. 2001).

### Immunohistochemistry

Immunohistochemistry (IHC) was conducted to localize microsystin within the fish tissue using techniques that we have previously validated and optimized for threadfin shad and microsystin from the San Francisco Estuary (Acuña et al. 2012a, 2012b). Formalin‐fixed whole threadfin shad samples (see previous *Fish collection* section) were embedded in paraffin, sectioned, and assessed by IHC for proteins associated with the presence of microsystin‐LR. The microsystin‐LR antibodies cross‐reacted with all the microsystins detected and were reported as microcystin. Gut and liver tissues of threadfin shad from the sites Sherman Island, Brannon Island, Mildred Island, and Stockton were assessed for the presence of microsystin‐LR using microsystin‐LR antibodies (Axxora Biochemicals). A 1:200 dilution for all antibodies was utilized in the immunoperoxidase reaction of the goat ABC staining system kit (Santa Cruz Biotechnology). A positive control included a serial section that stained positive for microsystin‐LR using a positive blank, whereas a negative control was treated with all the reagents in the goat ABC staining system without the microsystin‐LR primary antibody. The positive staining of the positive blank and the lack of staining for the negative control was used to verify the validity of the IHC method.

### Statistical analyses

Data were analyzed using JMP Ver 8.0 (SAS Institute). Analysis was conducted using one‐way analysis of variance (ANOVA) to determine the difference in morphological and nutritional parameters from fish collected at different locations. Tukey's multiple mean comparison tests were used to assess for significant differences (*p* < 0.05) between individual sites and between bloom and nonbloom sites. Differences in histopathology variables between bloom and nonbloom sites were determined by the nonparametric comparison test analysis of similarities using PRIMER‐e statistical software (Clarke and Gorley 2015). The assumptions of normality for ANOVA were tested using Shapiro–Wilk and Levene tests. Data are presented as mean ± standard deviation (SD).

## RESULTS

### *Microcystis* and concentrations of microcystin in ambient water

Cyanobacterial blooms comprised of *Microcystis* colonies were observed during sampling at Brannon Island and Mildred Island, but there were no visible signs of *Microcystis* colonies at Sherman Island and Stockton. The California Department of Fish and Wildlife survey data as part of the Interagency Ecological Study Program (2019) monitoring confirmed the absence or low abundance of *Microcystis* colonies at Sherman Island and Stockton using the index they had developed for visual ranking of algal colonies on surface waters. *Microcystis* colonies on the surface of the water column at Sherman Island were ranked as 0 during August and September and at Stockton as 2 and 0 during August and September, respectively. Rank 0 represented no bloom detected, and rank 2 represented some colonies detected. Median *Microcystis* abundance and the associated total microsystins in August and September were highest in the San Joaquin River at Antioch and Franks Tract, in the upper San Francisco Estuary at Mildred Island and north of the confluence in the Sacramento River at Brannon Island. The average total microsystin concentration of *Microcystis* colonies during collection was 0.007 ± 0.000 and 0.032 ± 0.000 µg/L for Brannon Island and Mildred Island, respectively. No *Microcystis* colonies were detected on surface waters at Sherman Island and Stockton during fish collection, and therefore no microsystin determinations were conducted.

### Morphometric indices analysis

**Figure 2:**
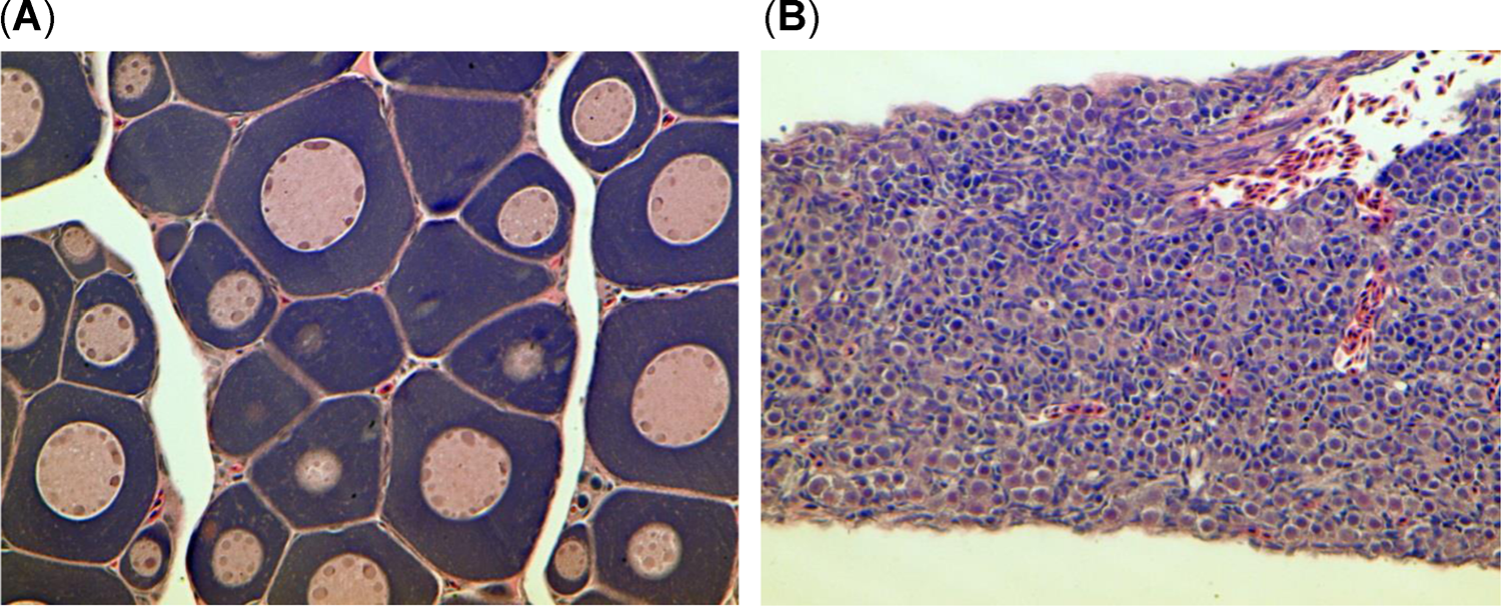
Gonadal tissue from treadfin shad showing (**A**) immature oogonia and (**B**) immature spermatogonia.

Despite multiple attempts to collect enough threadfin shad for all the metrics, there were only enough samples for all the metrics for Sherman Island, Stockton, and Brannon Island, and insufficient samples were obtained for nutritional indices for the Mildred Island site. Morphometric data are reported in Table 2. The *W*_b_ and fork length of threadfin shad were variable and unrelated to whether they were from nonbloom (Sherman Island and Stockton) or bloom sites (Brannon Island and Mildred Island). The threadfin shad collected at Mildred Island had the shortest fork length and the smallest *W*_b_ compared with those collected from the other 3 sites (*p* < 0.05). The threadfin shad from Brannon Island were shown to have the highest *W*_b_ and fork length, which were similar to those of threadfin shad from Stockton but were higher than those from Sherman Island (*p* < 0.05). Gonadal tissue was not readily detected by necropsy and when occasionally detected by histology was at an immature stage (Figure 2); therefore HSI and condition factor were not differentiated by sex. Like *W*_b_ and fork length, the general health indices of HSI and condition factor were variable and unrelated to whether they were from threadfin shad from the nonbloom or bloom sites. Significant differences in fork length may have affected the comparison. A comparison of condition factors for threadfin shad that were not significantly different by fork length showed that threadfin shad from the nonbloom site, Stockton exhibited elevated condition factor compared with threadfin shad from the bloom site, Brannon Island. Morphometric raw data are included in the Supplemental Data (Table S1).

**Table 2:** Morphometric indices (mean ± SD) for threadfin shad, *Dorosoma petenense*, collected from 4 study sites in the upper San Francisco Estuary during August and September 2007^a^

Location	Fork length (cm)	*W*_b_ (g)	HSI	Condition factor
Sherman Island	8.62 ± 0.61 B	7.42 ± 2.46 B	0.56 ± 0.18 AB	0.98 ± 0.13 B
Stockton	8.73 ± 0.80 AB	7.90 ± 2.40 AB	0.47 ± 0.13 B	1.15 ± 0.23 A
Brannon Island	9.95 ± 1.34 A	10.28 ± 4.39 A	0.52 ± 0.15 B	0.98 ± 0.12 B
Mildred Island	4.78 ± 0.64 C	1.37 ± 0.25 C	0.77 ± 0.21 A	1.33 ± 0.45 A

^a^ Significant differences between sites are indicated by different letters using Tukey's multiple means comparison test (*p* < 0.05).

SD = standard deviation; *W*_b_ = body weight; HIS = hepatosomatic index.

### Histopathology of threadfin shad from different collection sites

**Figure 3:**
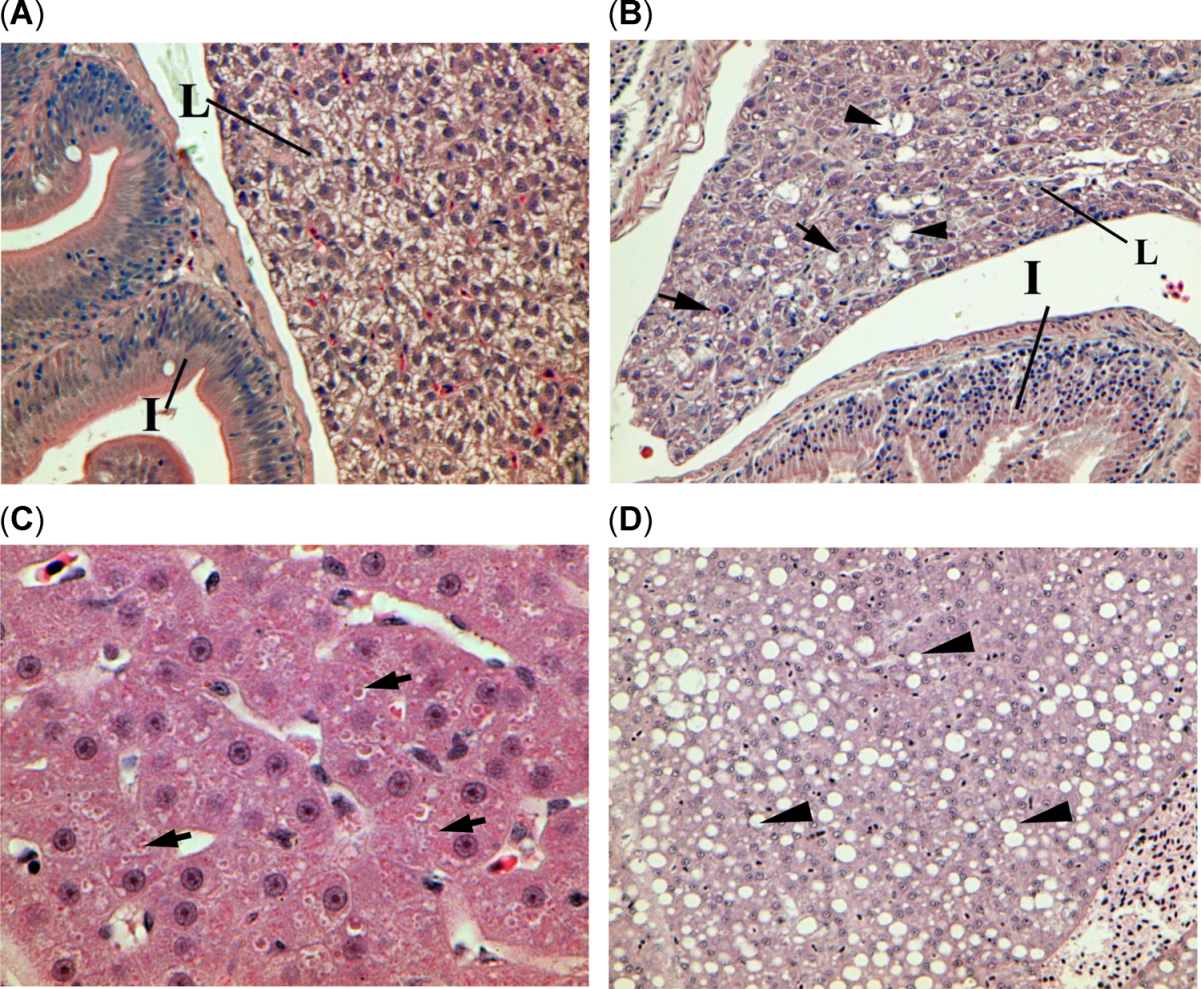
Histopathological sections of threadfin shad, *Dorosoma petenense*, (**A**) from the nonbloom site Sherman Island showing normal intestine (I) and glycogen‐rich liver (L) at ×100, (**B**) from the bloom site Mildred Island showing glycogen depletion (reduced prevalence of irregular shaped unstained tissue), single cell necrosis (arrows), and lipidosis (arrowheads) at ×100, (**C**) from the nonbloom site Stockton showing severe glycogen depletion and cytoplasmic inclusion bodies (arrows) at ×400, and (**D**) from the bloom site Brannon Island showing severe lipidosis (arrowheads) at ×200.

**Figure 4:**
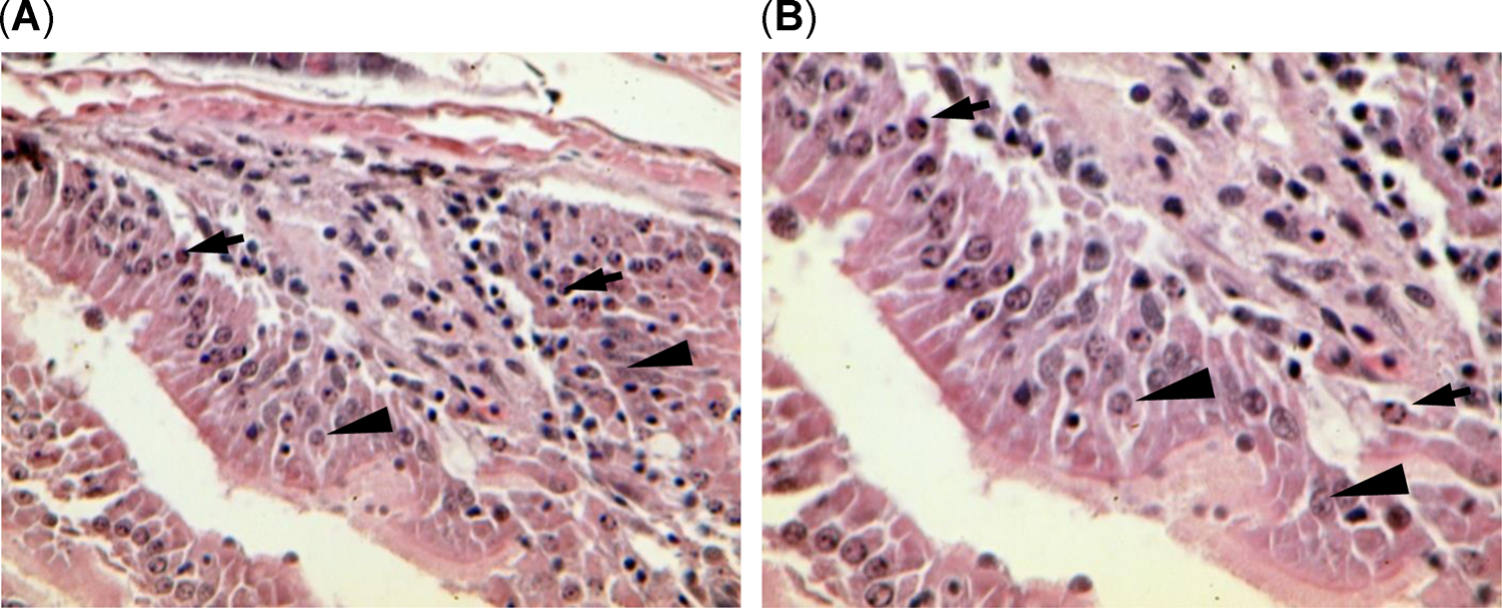
The gastrointestinal tract of threadfin shad, *Dorosoma petenense*, from bloom site Brannon Island also showed severe epithelial cell necrosis (karyopyknosis and karyorrhexis) at (**A**) ×400 and (**B**) ×600, indicating potential exposure to toxin. Arrows point to karyopyknosis and karyorrhexis, and arrowheads point to normal enterocytes.

Histopathological examination of liver detected cell damage and glycogen depletion from each site (Table 3). Raw data are reported in the Supplemental Data, Table S2. Liver lesions of lipidosis and single‐cell necrosis, as shown in Figure 3, tended to be detected in threadfin shad with greater severity in the bloom sites (Brannon Island and Mildred Island), whereas lesions of cytoplasmic inclusion bodies (Figure 3C) were detected in greater severity in the nonbloom sites (Sherman Island and Stockton). Glycogen depletion was significantly more severe in the bloom sites than the nonbloom site of Sherman Island but were similar in threadfin shad from Stockton. In addition, severe necrosis of the gut mucosa was observed in threadfin shad from the bloom site, Brannon Island (Figure 4).

**Table 3:** Mean histopathological lesion scores (±SD) for the liver of threadfin shad, *Dorosoma petenense*, collected from 4 study sites in the upper San Francisco Estuary during August and September 2007^a^

Sites	GD	MA	LIP	EDP	INF	CIB	SC	SCN
Sherman Island^b^	0.39 ± 0.66 C	0.07 ± 0.17 A	0.13 ± 0.46 AB	0.00 ± 0.00 A	0.28 ± 0.52 A	0.09 ± 0.42 B	0.00 ± 0.00 A	0.02 ± 0.10 B
Stockton^b^	2.10 ± 0.79 A	0.13 ± 0.28 A	0.05 ± 0.22 B	0.00 ± 0.00 A	0.30 ± 0.41 A	1.33 ± 0.95 A	0.00 ± 0.00 A	0.08 ± 0.24 B
Brannon Island	1.53 ± 1.07 B	0.12 ± 0.33 A	0.94 ± 1.03 A	0.00 ± 0.00 A	0.29 ± 0.47 A	0.00 ± 0.00 B	0.00 ± 0.00 A	0.59 ± 0.87 A
Mildred Island	2.47 ± 0.52 A	0.17 ± 0.31 A	0.20 ± 0.37 A	0.00 ± 0.00 A	0.10 ± 0.21 A	0.00 ± 0.00 B	0.00 ± 0.00 A	0.07 ± 0.18 AB

^a^ Significant differences using the nonparametric comparison test analysis of similarities (ANOSIM; *p* < 0.05) are indicated by uppercase letters.

^b^ Sites with no *Microcystis* blooms detected during sampling.

SD = standard deviation; GD = glycogen depletion; MA = macrophage aggregate; LIP = lipidosis; EDP = eosinophilic droplets; INF = infiltration of inflammatory cells; CIB = cytoplasmic inclusion bodies; SC = sinusoidal congestion; SCN = single‐cell necrosis.

### Occurrence of *Microcystis* and microcystin‐LR in threadfin shad

**Figure 5:**
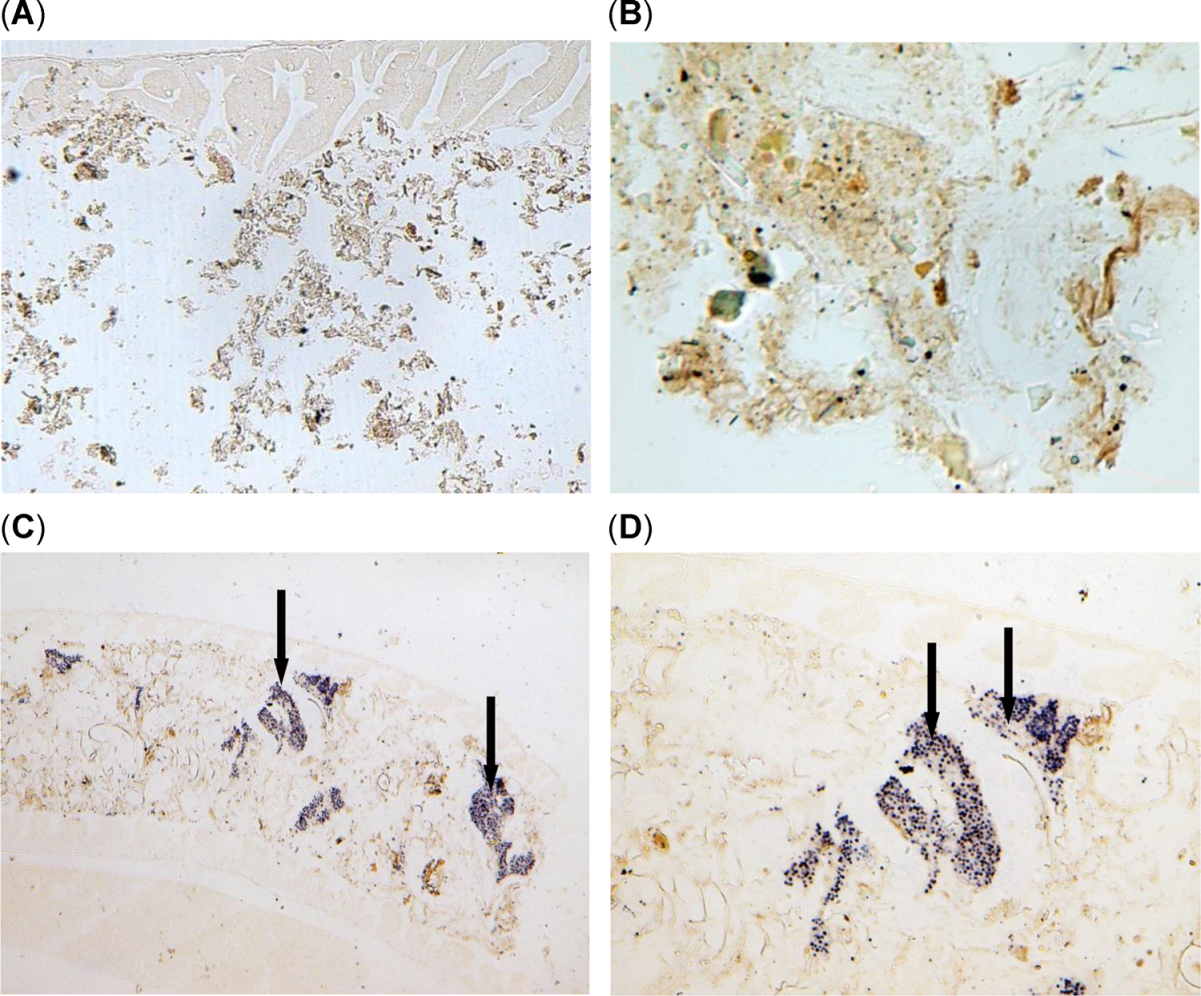
In situ hybridization of the intestine of threadfin shad, *Dorosoma petenense*, from the upper San Francisco Estuary. A negative staining of *Microcystis* DNA in the intestine of threadfin shad from Sherman Island with no *Microcysti*s at (**A**) ×100 and (**B**) ×400. Blue precipitates indicate ingested *Microcystis* cells (arrows) in the lumen of the intestine and in between the intestinal wall and lining of threadfin shad at (**C**) ×100 and (**D**) ×400 from bloom‐impacted Mildred Island.

Using the ISH assay already validated in previous laboratory exposures to *Microcystis* (Acuña et al. 2012a), localizations of *Microcystis* DNA (purple colorations) in gastrointestinal tracts of threadfin shad from Mildred Island are shown in Figure 5. The following results confirm that the ISH assay is valid: 1) lack of hybridizations signals in gut contents without *Microcystis* when hybridized with the labeled probe (Figure 5A,B); 2) presence of hybridization signals in stomach and gut contents of threadfin shad with *Microcystis* cells after treatment with the labeled probe (Figure 5C,D); and 3) lack of signals in *Microcystis‐*positive threadfin shad tissues after treatment with unlabeled probes (data not shown). The presence of *Microcystis* DNA in stomach contents and adjacent intestinal lining of threadfin shad from the bloom sites, Brannon Island and Mildred Island (Figure 5C,D) confirms direct ingestion of *Microcystis* by threadfin shad.

**Figure 6:**
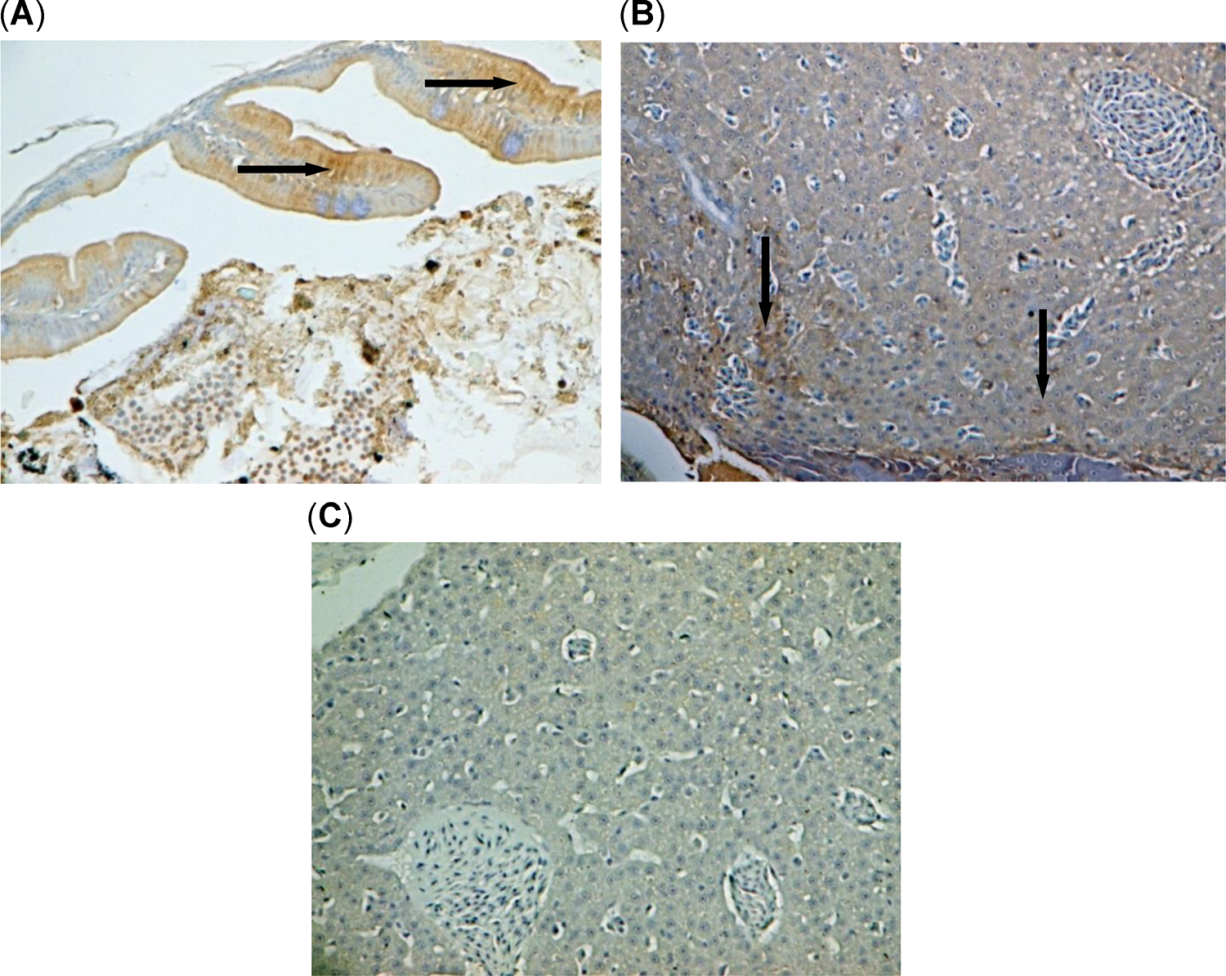
Microcystin‐LR localization as shown in positive brownish stains (arrows) in (**A**) intestine at ×400 and (**B**) liver of threadfin shad (*Dorosoma petenense*) at ×100, from Brannon Island with *Microcystis* blooms compared with liver of threadfin shad from Sherman Island without *Microcystis* bloom, which did not show any positive staining with microcystin‐LR in liver tissue (**C**) at ×100.

The IHC analysis for microcystin‐LR also indicated the accumulation of microsystin toxin in the gut and liver of threadfin shad from the bloom sites Brannon Island and Mildred Island, where both stations had abundant *Microcystis* colonies on the surface of the water column (Figure 6). These sections did not show any positive signals when treated with unlabeled microsystin‐LR probes, confirming that the IHC assay was valid in localizing microsystin‐LR in gut and liver of threadfin shad. The IHC did not detect the presence of microsystins in threadfin shad from the nonbloom sites Sherman Island (Figure 6C) and Stockton, where *Microcystis* colonies were lacking on surface waters during sampling.

### Nutritional composition of threadfin shad

The nutritional indices by proximate analysis and RNA‐to‐DNA ratio determination of the fish were contrasted statistically between sites. For the proximate analysis there was insufficient sample allocated from the bloom site Mildred Island, and therefore Mildred Island was precluded from the statistical analysis. Preservation of samples in ethanol in formalin reduced the number of fish available for the proximate analysis for all sites. Although there were sufficient samples for the proximate analysis for Sherman Island, Brannon Island, and Stockton, there was insufficient sample (*n* = 1) for Mildred Island. Results from the proximate analyses and RNA‐to‐DNA ratio are shown in Tables 4 and 5, respectively. The moisture content, protein content, and lipid content in fish tissue were variable and unrelated to the presence or absence of the *Microcystis* bloom. The moisture content was the highest and lipid content was the lowest in threadfin shad collected from Stockton. The threadfin shad collected from Brannon Island tended to have the highest protein and lipid contents, and these contents were significantly higher than then threadfin shad from Stockton (*p* < 0.05). The highest RNA‐to‐DNA ratios were measured in threadfin shad collected at the nonbloom site Sherman Island and the bloom site Mildred Island (Table 5). Raw data on proximate analysis are given in the Supplemental Data (Table S3). The raw data on the RNA‐to‐DNA ratios are also given in the Supplemental Data (Table S1).

**Table 4:** Nutritional indices (mean ± SD) of percentages of moisture, protein, and lipid measured for threadfin shad, *Dorosoma petenense*, collected from the 4 study sites in the upper San Francisco Estuary during August and September 2007^a^

Location	Moisture	Protein	Lipid
Sherman Island	75.29 ± 1.87 B	15.25 ± 0.44 AB	2.43 ± 0.56 A
Stockton	78.51 ± 0.69 A	14.76 ± 0.44 B	1.06 ± 0.15 B
Brannon Island	74.52 ± 2.76 B	15.85 ± 0.20 A	3.32 ± 3.22 AB
Mildred Island	79.96^b^	14.94^b^	0.43^b^

^a^ Significant differences using Tukey's multiple mean comparison tests for all the nutritional indices for all sites except Mildred Island (due to insufficient sample) are indicated by different letters (*p* < 0.05).

^b^ Insufficient number of sample to perform replicates.

SD = standard deviation.

**Table 5:** Mean (±SD) of the RNA‐to‐DNA ratio in the muscle tissue of threadfin shad, *Dorosoma petenense*, collected from the 4 study sites in the upper San Francisco Estuary during August and September 2007^a^

Location	No.	Average
Sherman Island^b^	10	3.53 ± 0.84 A
Stockton^b^	8	2.25 ± 0.85 C
Brannon Island	10	2.61 ± 0.77 B
Mildred Island	8	3.86 ± 0.81 A

^a^ Significant differences using Tukey's multiple mean comparison tests between all sites for RNA‐to‐DNA ratios are indicated by different letters (*p* < 0.05).

^b^ Sites with no *Microcystis* blooms detected during sampling.

SD = standard deviation.

## DISCUSSION

The results of the ISH and IHC analyses of *Microcystis* and microsystins in threadfin shad confirms our hypothesis of dietary ingestion and digestion of *Microcystis* colonies in the field. The detection of *Microcystis* DNA in the gut (by ISH) confirms that threadfin shad are omnivorous (Turner 1966; Ingram and Ziebell 1983) and as indiscriminate particulate and filter feeders will consume *Microcystis* colonies when they are present. Although cyanobacteria have barriers to digestion such as thick cell walls, the presence of the microsystins within the gut and liver tissue by IHC suggests that threadfin shad were able to digest *Microcystis*, which would allow microsystins to enter the target organs. This direct ingestion and digestion of *Microcystis* colonies was found to lead to the release and incorporation of microsystins into target organs. Because the microcystin toxin was detected in the liver and gut tissue before the senescence of the bloom, there is a risk of toxicity to fish due to both dissolved and particulate sources of microcystin throughout the bloom and its senescence. A study by Carbis et al. (1997) found that more than half of the common carp analyzed after exposure to microsystin from *Microcystis* consumption exhibited liver lesions. Exposure to microsystins can result in impaired liver function (lipidosis) and increased single‐cell necrosis in the liver of brown trout, *Salmo trutta* (Bury et al. 1997), common carp (Carbis et al. 1996), and medaka, *O. latipes* (Deng et al. 2010). Lesions and necrotic cells were also observed in the liver of laboratory‐raised splittail after long‐term, low‐level exposure to dietary *Microcystis* in laboratory bioassays (Acuña et al. 2012a).

The indices of condition factor and HSI were variable with exposure to *Microcystis* and microcystin. Significant differences in size may explain the results, because threadfin shad from Brannon Island and Stockton tended to be bigger than threadfin shad from Sherman Island and Mildred Island. Size may significantly affect condition factor and HSI. By only comparing the condition factor and HSI from similarly sized fish from the nonbloom site, Stockton, and the bloom site, Brannon Island, it was determined that condition factor was significantly elevated in threadfin shad from Stockton. This finding suggests that exposure to the microcystin toxin, confirmed by bioassays for threadfin shad from bloom sites, may reduce their general health. Because only a limited number of threadfin shad could be contrasted this way, the results are only suggestive, and further field work would need to be conducted to confirm the relationship. Laboratory bioassays confirmed the negative correlation between *Microcystis* toxins and condition factor in threadfin shad of similar size at ambient concentrations in the upper San Francisco Estuary. The HSI values of threadfin shad exposed to high and environmentally relevant microcystin concentrations in the laboratory were not significantly different, suggesting that HSI is not a sensitive biomarker of exposure for threadfin shad (Acuña et al. 2012b) and that the differences detected in HSI in the present study may be related to other factors. The smaller size of threadfin shad from Mildred Island likely indicates the fish were younger, and the corresponding larger size of the HSI may be a result of increased somatic growth rates.

We could not determine a clear relationship between the presence of *Microcystis* in the gut contents and exposure of microcystin in the liver and gut tissue with nutritional status. Previous studies showed that exposure to microsystins may reduce nutritional status due to liver damage (Bury et al. 1996; Oberemm et al. 1997; Leao et al. 2009; Deng et al. 2010; Acuña et al. 2012a, 2012b), energy cost for detoxification (Pflugmacher et al. 1998; Prieto et al. 2006; Amado and Monserrat 2010), and reduced feeding (Beveridge et al. 1993). The disparity of results suggests that nutritional status may be sensitive to other predisposing factors (e.g., site specific, life stage specific, prey availability) that were not assessed in our study and were not directly correlated to presence and effect of the blooms.

Although direct ingestion and incorporation of the toxin microcystin was detected, the direct impact of microcystins on the histopathology of wild fish was not conclusive because the lesion scores did not all correlate with either bloom or nonbloom sites and were not specific to microcystin exposure. Elevated lesion scores for lipidosis and single‐cell necrosis tended to be observed in threadfin shad from the bloom sites Brannon Island and Mildred Island, but sinusoidal congestion was not detected in any of the threadfin shad, which suggests a potential toxicity effect from *Microcystis* and microcystin but not definitively. Elevated scores for lipidosis and single‐cell necrosis due to *Microcystis* and microcystin exposure were found in a laboratory diet exposure study with threadfin shad (Acuña et al. 2012b). Although sinusoidal congestion is a lesion common in fish with severe and long‐term exposure to *Microcystis* (Bury et al. 1997; Acuña et al. 2012b), the lack of sinusoidal congestion in threadfin shad from any of the sites makes it difficult to attribute the other lesions to exposure to the cyanobacteria bloom. Cytoplasmic inclusion bodies were another lesion that was elevated significantly in threadfin shad from Stockton but not in the bloom sites, suggesting there may be additional stressors in the environment. The great numbers of cytoplasmic inclusion bodies were correlated with reduced HSI, increase glycogen depletion, and significantly low RNA‐to‐DNA ratio, suggesting nutritional stress (Love 1970; Segner and Moller 1984). The upper San Francisco Estuary is an urbanized and agriculturally rich estuary and receives runoff and discharge from both urban and agricultural sources (Orlando et al. 2014; Fong et al. 2016). It is possible that the presence of other contaminants in the upper San Francisco Estuary may enhance liver damage to threadfin shad from *Microcystis* (Bailey et al. 2000; de Vlaming et al. 2000; Orlando 2013). Furthermore, fish exposure may vary due to the spatial and temporal variability of *Microcystis* blooms and fish movement. *Microcystis* colonies were detected on the surface of the water column at Stockton during routine Interagency Ecological Study Program (2019) monitoring several weeks before the fish collection. Liver lesions were measured in both inland silversides and juvenile striped bass collected at many locations throughout the San Francisco Estuary, even though *Microcystis* abundance was low (Lehman et al. 2010).

Severe necrosis was detected in the gastrointestinal tract of threadfin shad collected from the bloom site Brannon Island. The necrosis may have been due to *Microcystis* and microcystin toxicity from direct ingestion of *Microcystis* (Sivonen and Jones 1999) and/or a combination of multiple stressors present in the San Francisco Estuary (Brooks et al. 2012; Fong et al. 2016). The lipopolysaccharides of cyanobacterial cell walls can cause inflammatory response and oxidative stress, which can lead to cell necrosis (Ito et al. 1997; Sivonen and Jones 1999; Cox et al. 2005; Downing et al. 2011). The toxic effects of lipopolysaccharides and oxidative stress related to the metabolism of microsystins could have contributed to cell necrosis (Maher 2005; Prieto et al. 2006). Alternatively, gastrointestinal necrosis can be explained by the myriad number of other toxicants prevalent in the San Francisco Estuary (Brooks et al. 2012; Fong et al. 2016). The necrotic gut tissue is not typically associated with *Microcystis* exposure and is likely the result of other stressors.

*Microcystis* and its metabolite microsystin are potential stressors of fish in the San Francisco Estuary (Feyrer et al. 2009; Acuña et al. 2012b), yet it has not been established whether *Microcystis* is biologically available to fish. By employing a novel approach of combining various analytical tools and a common pelagic fish, threadfin shad, our study used multiple lines of evidence to determine whether exposure to *Microcystis*/microsystin during blooms was through a dietary exposure pathway. Although toxicity detected in the threadfin shad could not be attributed to exposure to *Microcystis* because there were likely multiple antecedent interactions associated with the study site being a highly altered estuary, our study showed that threadfin shad were directly exposed to the toxin microcystin from *Microcystis* blooms throughout the bloom season and not just during the subsequent senescence when microsystins would be released from lysed cells. *Microcystis* blooms are an increasing concern globally as well as in the San Francisco Estuary, with increased trajectory projections in the frequency and severity of *Microcystis* blooms in relation to climate change (Intergovernmental Panel on Climate Change 2014; Dettinger et al. 2016; Lehman et al. 2017). The novel approach detailed in our study can be modified to determine dietary exposure of *Microcystis* to fish. Identifying other potential stressors present in the San Francisco Estuary in addition to dietary exposure to *Microcystis* and microsystin toxicity will be an important next step needed when important measures for future fishery management are implemented.

## Supplemental Data

The Supplemental Data are available on the Wiley Online Library at DOI: https://doi.org/10.1002/etc.4659.

## Supporting information

This article includes online‐only Supplemental Data.

Supporting information.

Supporting information.

Supporting information.

Supporting information.

Supporting information.

Supporting information.

Supporting information.

## Data Availability

Data pertaining to this manuscript are attached as Supplemental Data.
