# Supplementary figures and images for: Determining the Exposure Pathway and Impacts of Microcystis on Threadfin Shad, Dorosoma petenense, in San Francisco Estuary
Source: Environ Toxicol Chem. 2020 Feb 21;39(4):787–98. doi: 10.1002/etc.4659 (PMC7155034; doi:10.1002/etc.4659)

Fig. S1a

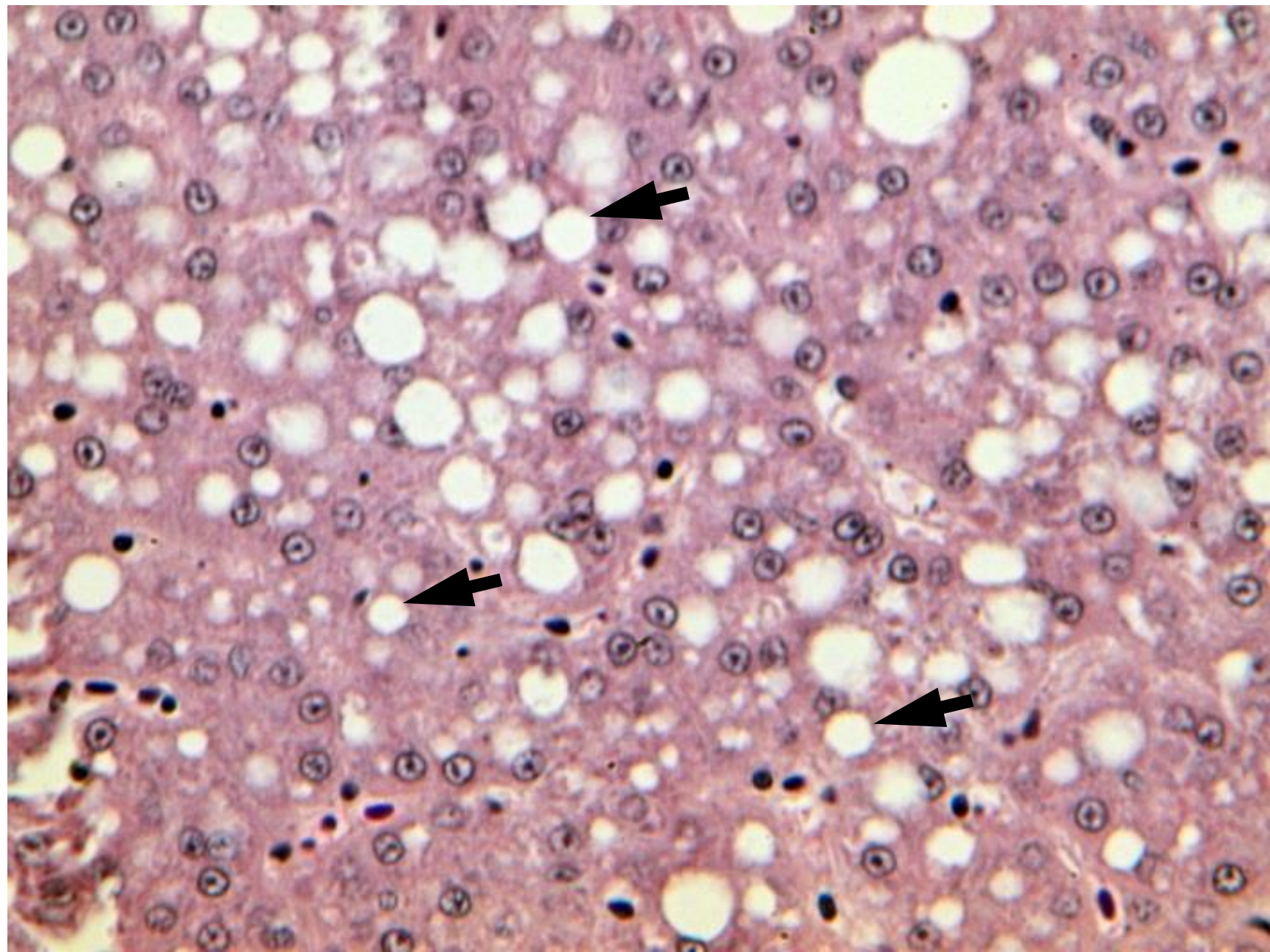

Supplement: Supplementary file 1 — Supporting information. [file ETC-39-787-s001.pdf]

Fig. S1b

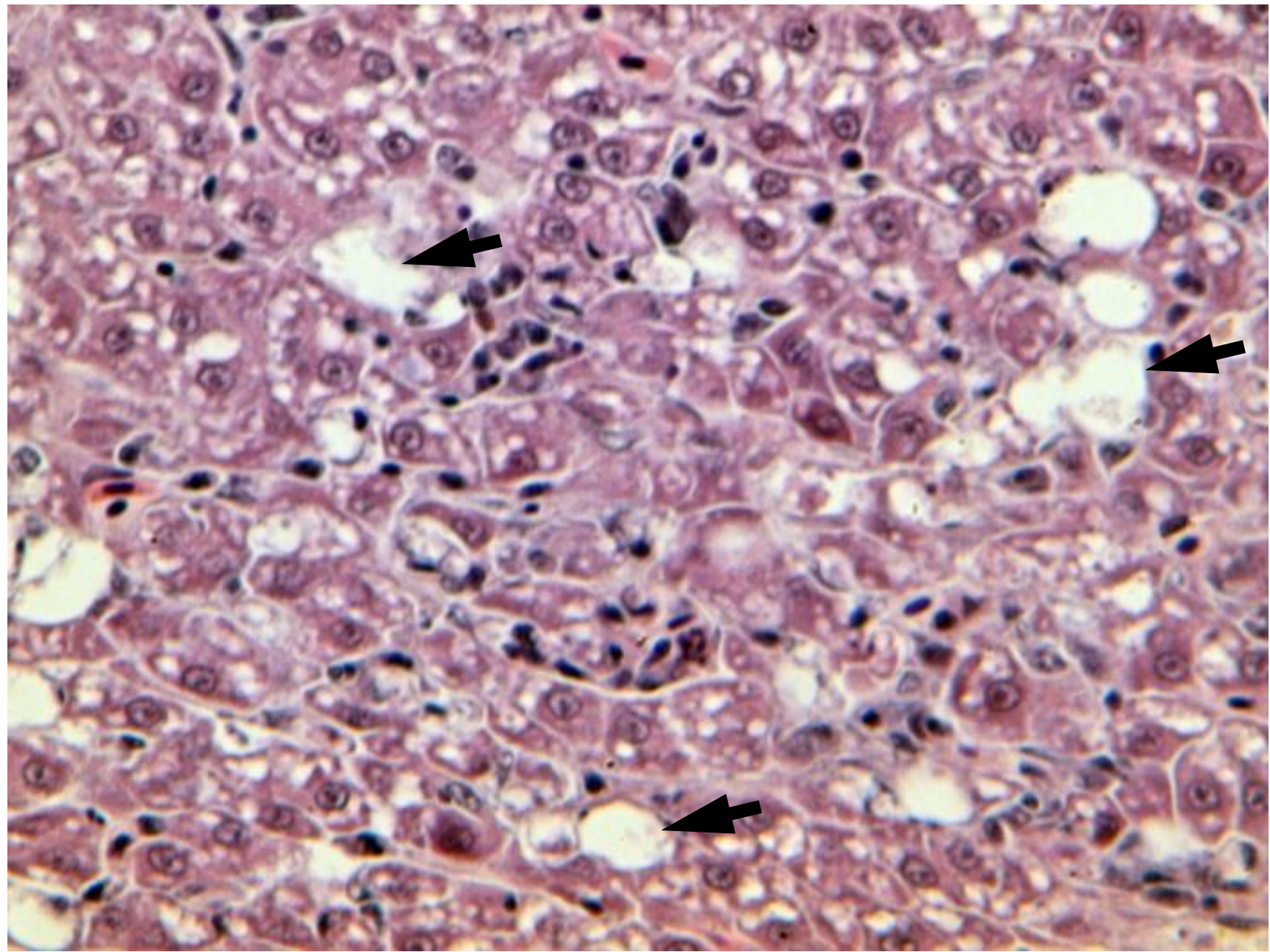

Supplement: Supplementary file 2 — Supporting information. [file ETC-39-787-s002.pdf]

Fig. S2a

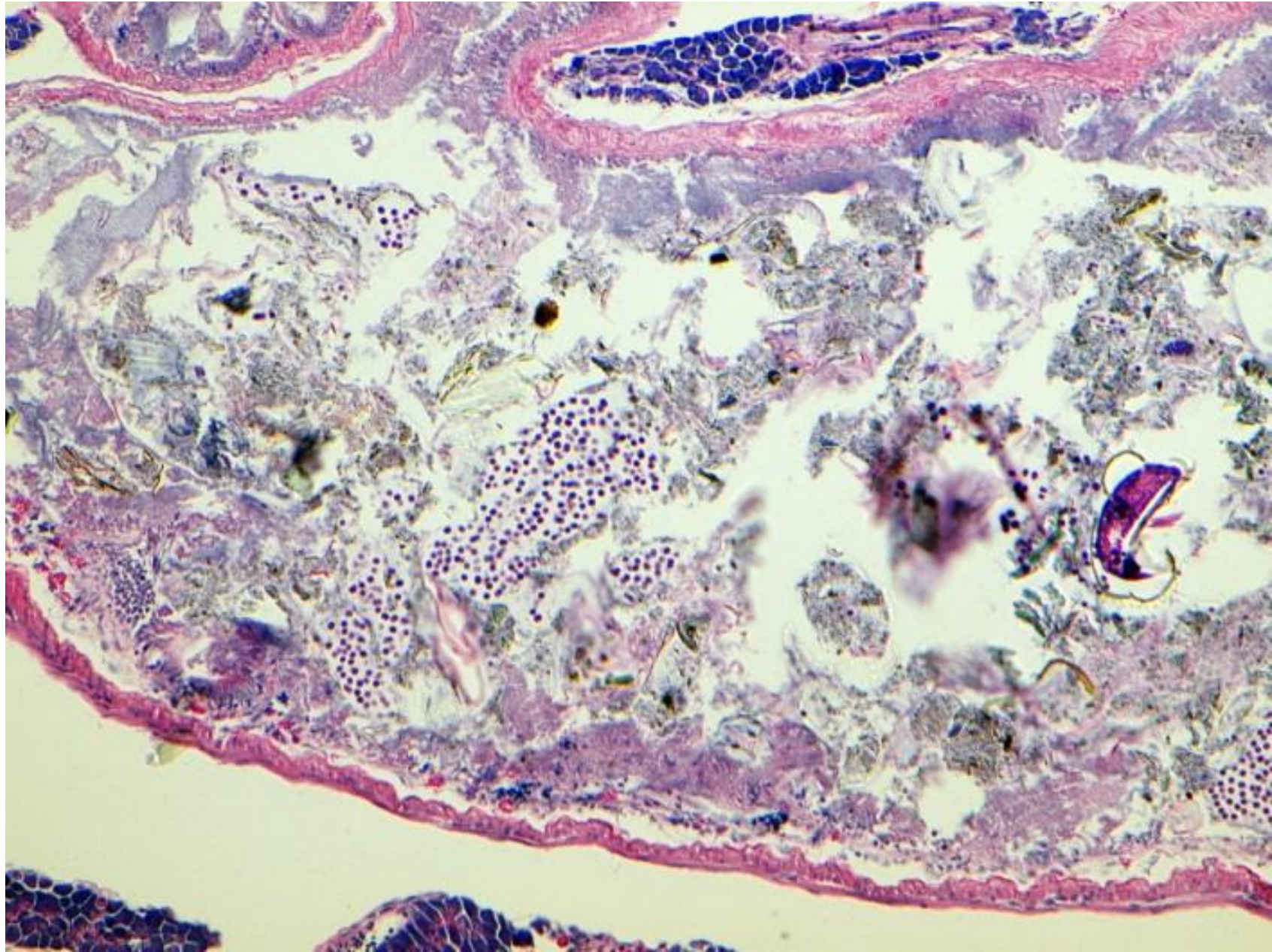

Supplement: Supplementary file 3 — Supporting information. [file ETC-39-787-s003.pdf]

Fig. S2b

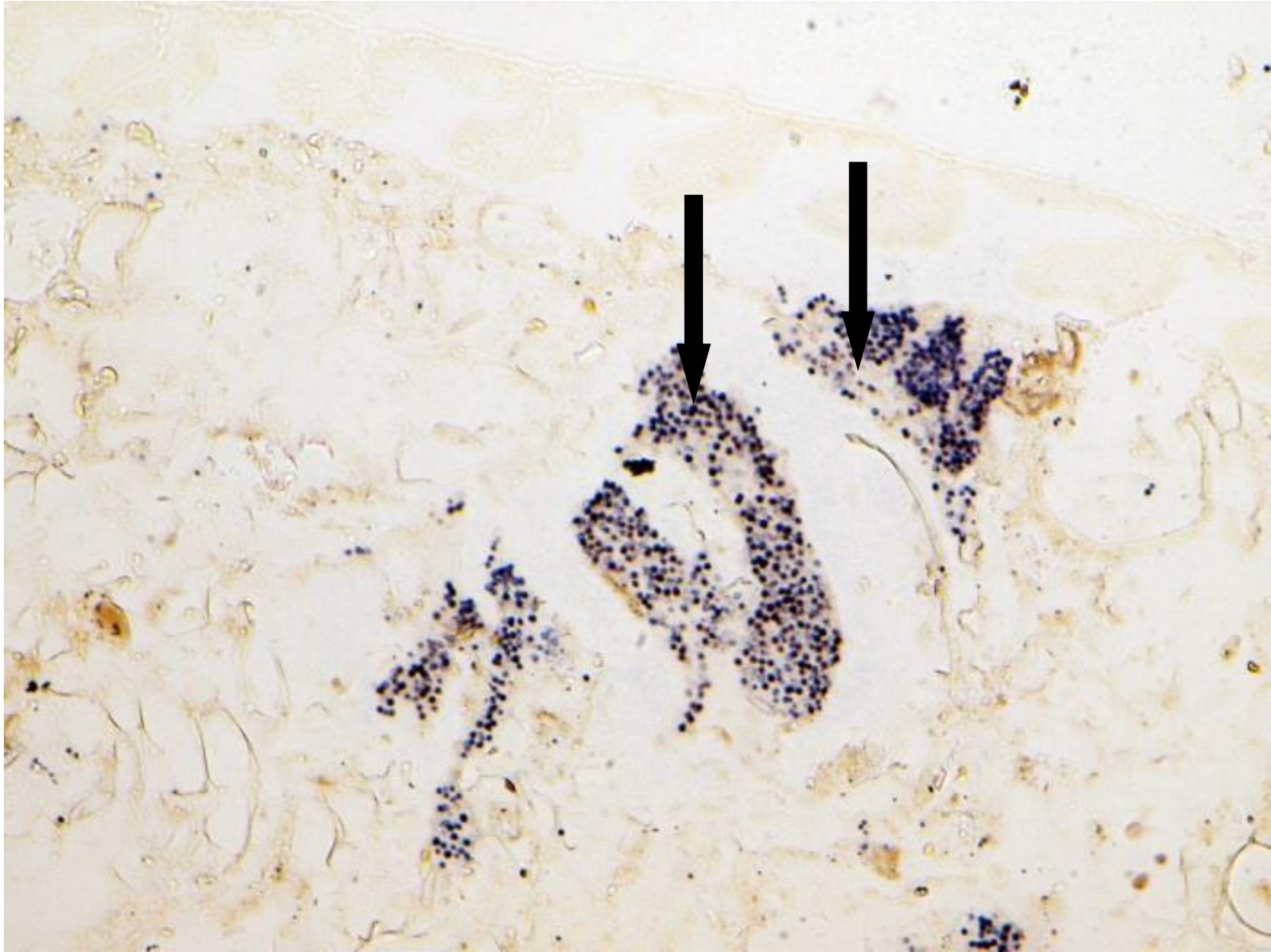

Supplement: Supplementary file 4 — Supporting information. [file ETC-39-787-s004.pdf]

Fig. S3a

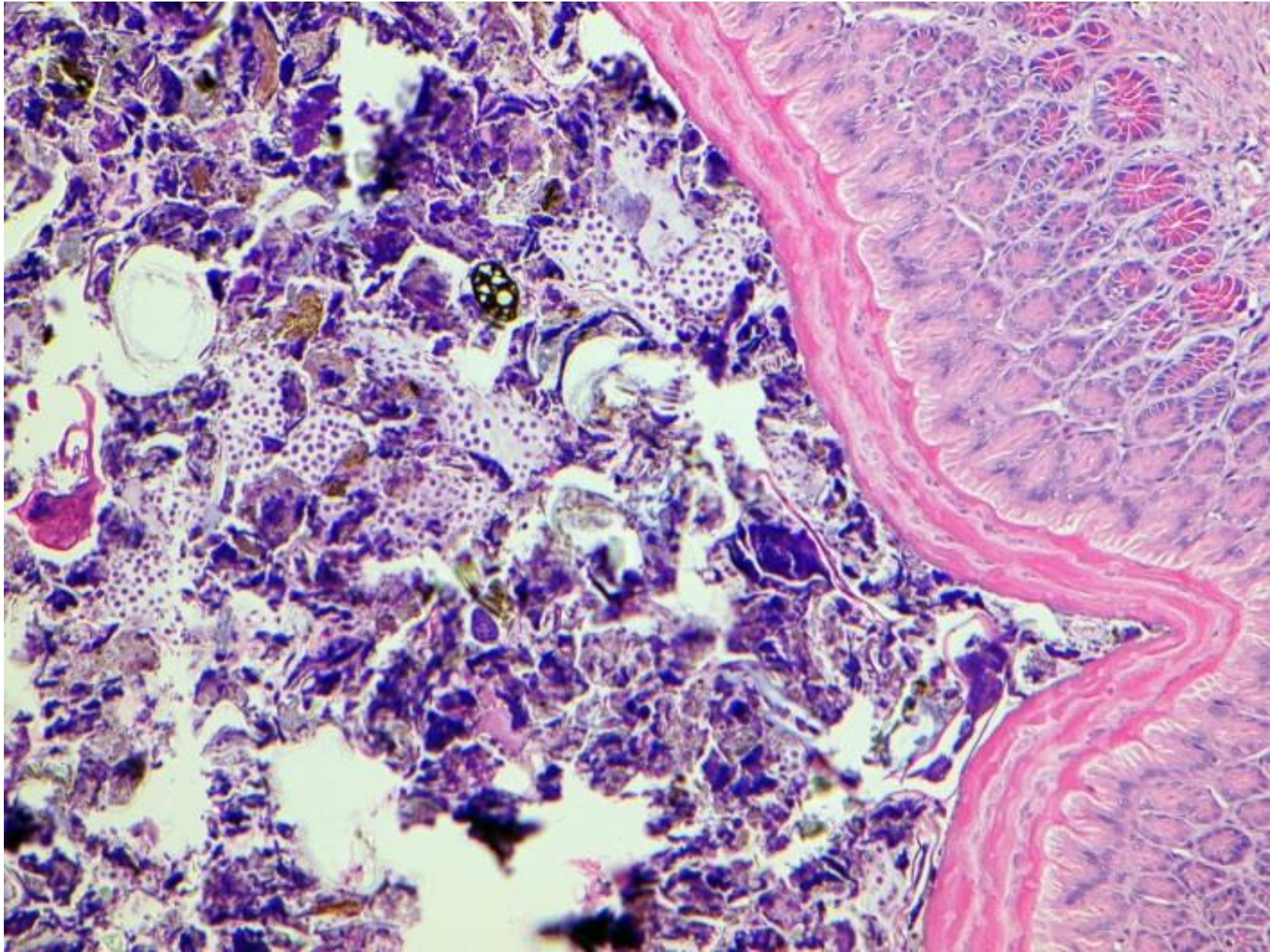

Supplement: Supplementary file 5 — Supporting information. [file ETC-39-787-s005.pdf]

Fig. S3b

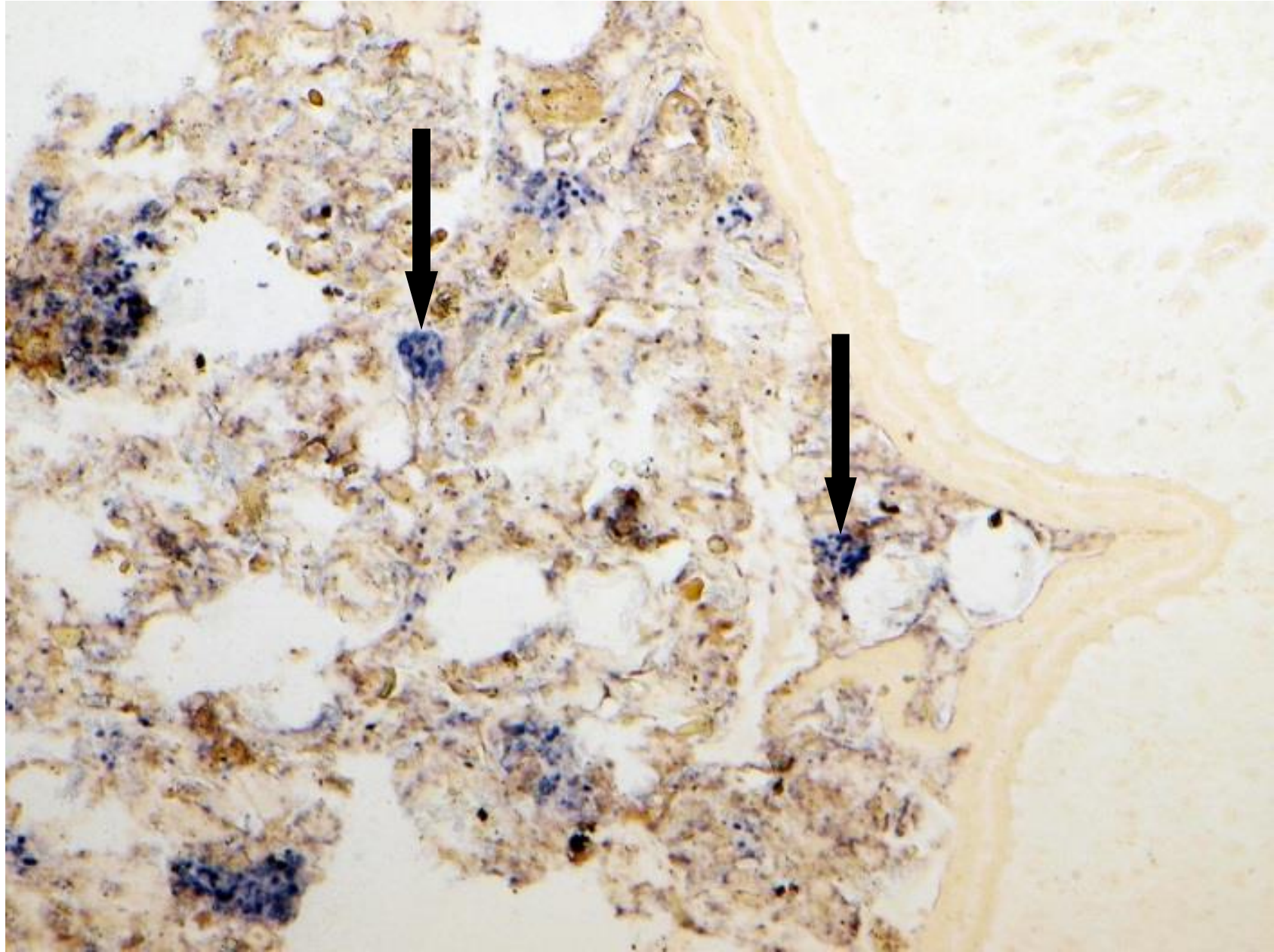

Supplement: Supplementary file 6 — Supporting information. [file ETC-39-787-s006.pdf]
