# Supplementary material for: Determining the Exposure Pathway and Impacts of Microcystis on Threadfin Shad, Dorosoma petenense, in San Francisco Estuary
Source: Environ Toxicol Chem. 2020 Feb 21;39(4):787–98. doi: 10.1002/etc.4659 (PMC7155034; doi:10.1002/etc.4659)
Supplement: Supplementary file 7 — Supporting information. [file ETC-39-787-s007.docx]

**Supplementary Materials:**

**Title:** Determining the exposure pathway and impacts of *Microcystis* on Threadfin shad, *Dorosoma petenense*, in San Francisco Estuary

**Journal:** Environmental Toxicology and Chemistry

**Authors:** Shawn Acuña^*^, Dolores Baxa, Peggy Lehman, Foo-Ching Teh, Dong-Fang Deng, Swee Teh

^*^ Metropolitan Water District of Southern California, 1121 L Street, Sacramento, CA, 95814; sacuna@mwdh2o.com; phone: (916) 650-2664, Corresponding author

Table S1. Fork Lenth (FL), body weight (W_b_), condition factor (CF), and RNA/DNA ratio measured for Threadfin shad collected at Sherman Island (SI), Mildred Island (MI), Brannan Island (BI), and Stockton (STK). N/A represent data that were not available.

| **Site** | **FL (cm)** | **W_b_(g)** | **W_l_(g)** | **CF（g/cm3)** | **HSI** | **RNA/DNA** |
| --- | --- | --- | --- | --- | --- | --- |
| SI | 8.00 | 4.33 | 0.014 | 0.85 | 0.33 | 3.04 |
| SI | 9.00 | 6.61 | 0.031 | 0.91 | 0.47 | 3.65 |
| SI | 8.50 | 5.99 | 0.012 | 0.98 | 0.21 | 2.77 |
| SI | 8.20 | 5.29 | 0.012 | 0.96 | 0.22 | 3.76 |
| SI | 8.80 | 6.41 | 0.034 | 0.94 | 0.53 | 4.70 |
| SI | 9.00 | 7.27 | 0.030 | 1.00 | 0.41 | 4.03 |
| SI | 8.20 | 4.40 | 0.028 | 0.80 | 0.63 | 3.65 |
| SI | 8.00 | 4.62 | 0.023 | 0.90 | 0.50 | 3.81 |
| SI | 10.00 | 11.30 | 0.078 | 1.13 | 0.69 | 1.69 |
| SI | 8.50 | 5.55 | 0.033 | 0.90 | 0.60 | 4.17 |
| SI | 9.70 | 11.43 | 0.067 | 1.25 | 0.59 | N/A |
| SI | 9.50 | 8.84 | 0.055 | 1.03 | 0.63 | N/A |
| SI | 9.00 | 9.74 | 0.060 | 1.34 | 0.62 | N/A |
| SI | 8.50 | 5.86 | 0.043 | 0.95 | 0.73 | N/A |
| SI | 9.10 | 6.69 | 0.030 | 0.89 | 0.45 | N/A |
| SI | 8.70 | 6.37 | 0.052 | 0.97 | 0.82 | N/A |
| SI | 10.80 | 12.38 | 0.117 | 0.98 | 0.95 | N/A |
| SI | 9.30 | 7.21 | 0.047 | 0.90 | 0.65 | N/A |
| SI | 10.40 | 10.43 | 0.055 | 0.93 | 0.53 | N/A |
| SI | 9.20 | 7.75 | 0.047 | 1.00 | 0.61 | N/A |
| BI | 9.40 | 9.87 | 0.030 | 1.19 | 0.30 | 2.08 |
| BI | 7.80 | 4.25 | 0.024 | 0.90 | 0.57 | 2.61 |
| BI | 7.80 | 4.22 | 0.021 | 0.89 | 0.50 | 1.75 |
| BI | 10.50 | 11.42 | 0.044 | 0.99 | 0.39 | 3.08 |
| BI | 10.30 | 9.85 | 0.038 | 0.90 | 0.39 | 3.04 |
| BI | 8.70 | 5.35 | 0.026 | 0.81 | 0.49 | 2.66 |
| BI | 9.20 | 6.61 | 0.042 | 0.85 | 0.64 | 1.97 |
| BI | 9.80 | 8.63 | 0.032 | 0.92 | 0.37 | 3.62 |
| BI | 10.20 | 11.07 | 0.062 | 1.04 | 0.56 | 3.67 |
| BI | 13.00 | 19.45 | 0.071 | 0.89 | 0.37 | 2.99 |
| BI | 10.50 | 12.20 | 0.065 | 1.05 | 0.53 | 1.27 |
| BI | 11.20 | 15.53 | 0.119 | 1.11 | 0.77 | N/A |
| BI | 10.00 | 11.00 | 0.048 | 1.10 | 0.44 | N/A |
| BI | 9.70 | 8.54 | 0.068 | 0.94 | 0.80 | N/A |
| BI | 11.20 | 16.23 | 0.108 | 1.16 | 0.67 | N/A |
| MI | 5.30 | 1.81 | 0.013 | 1.22 | 0.73 | N/A |
| MI | 4.80 | 1.30 | 0.015 | 1.18 | 1.18 | 5.21 |
| MI | 4.80 | 1.34 | 0.010 | 1.21 | 0.75 | 4.57 |
| MI | 3.90 | 1.39 | 0.011 | 2.34 | 0.80 | 3.98 |
| MI | 6.00 | 1.68 | 0.007 | 0.78 | 0.42 | 2.85 |
| MI | 4.50 | 1.11 | 0.008 | 1.22 | 0.72 | 3.54 |
| MI | 4.50 | 1.21 | 0.010 | 1.33 | 0.83 | 3.67 |
| MI | 4.40 | 1.15 | 0.008 | 1.35 | 0.70 | 3.20 |
| STK | 9.40 | 9.47 | 0.046 | 1.14 | 0.49 | 4.23 |
| STK | 8.30 | 6.84 | 0.036 | 1.20 | 0.53 | 2.24 |
| STK | 9.20 | 10.50 | 0.034 | 1.35 | 0.32 | 1.62 |
| STK | 9.50 | 6.68 | 0.045 | 0.78 | 0.67 | 1.67 |
| STK | 8.70 | 9.07 | 0.028 | 1.38 | 0.31 | 2.14 |
| STK | 8.90 | 8.69 | 0.036 | 1.23 | 0.41 | 2.06 |
| STK | 8.80 | 9.05 | 0.055 | 1.33 | 0.61 | 2.31 |
| STK | 7.00 | 2.87 | 0.012 | 0.84 | 0.42 | 1.70 |

**Table S2**. Histopathology score for liver lesions of Threadfin shad collected from Sherman Island (SI), Brannon Island (BI), Mildred Island (MI), and Stockton (STK). Lesions scored from 0-3 for glycogen depletion (GD), macrophage aggregate (MA), lipidosis (LIP), eosinophilic droplets (EDP), infiltration of inflammatory cells (INF), cytoplasmic inclusion bodies (CIB),sinusoidal congestion (SC) and single cell necrosis (SCN). Histopathological lesion scoring criteria:  0 = none/minimal, 1 = mild, 2 = moderate, and 3 = severe as assessed using light microscopy.

| Site | Date Collected | **GD** | **MA** | **LIP** | **EDP** | **INF** | **CIB** | **SC** | **SCN** |
| --- | --- | --- | --- | --- | --- | --- | --- | --- | --- |
| SI | 8/28/2007 | 0 | 0.5 | 0 | 0 | 0.5 | 0 | 0 | 0 |
| SI | 8/28/2007 | 0 | 0 | 0 | 0 | 0 | 0 | 0 | 0 |
| SI | 8/28/2007 | 0 | 0 | 0 | 0 | 0 | 0 | 0 | 0 |
| SI | 8/28/2007 | 1 | 0 | 0 | 0 | 1 | 0 | 0 | 0 |
| SI | 8/28/2007 | 0 | 0.5 | 0 | 0 | 0.5 | 0 | 0 | 0 |
| SI | 8/28/2007 | 1 | 0 | 0 | 0 | 1 | 0 | 0 | 0 |
| SI | 8/28/2007 | 0 | 0 | 0 | 0 | 0 | 0 | 0 | 0 |
| SI | 8/28/2007 | 0 | 0 | 0 | 0 | 0 | 0 | 0 | 0 |
| SI | 8/28/2007 | 2 | 0 | 0 | 0 | 0 | 0 | 0 | 0.5 |
| SI | 8/28/2007 | 0 | 0 | 0 | 0 | 0 | 0 | 0 | 0 |
| MI | 8/29/2007 | 3 | 0 | 0 | 0 | 0.5 | 0 | 0 | 0 |
| MI | 8/29/2007 | 3 | 0 | 0 | 0 | 0 | 0 | 0 | 0 |
| MI | 8/29/2007 | 2 | 0 | 0 | 0 | 0 | 0 | 0 | 0 |
| MI | 8/29/2007 | 2 | 0.5 | 0 | 0 | 0 | 0 | 0 | 0.5 |
| MI | 8/29/2007 | 2 | 0 | 0 | 0 | 0 | 0 | 0 | 0 |
| MI | 8/29/2007 | 2 | 0 | 0.5 | 0 | 0 | 0 | 0 | 0 |
| MI | 8/29/2007 | 3 | 0.5 | 0 | 0 | 0 | 0 | 0 | 0.5 |
| MI | 8/29/2007 | 2 | 0 | 0 | 0 | 0 | 0 | 0 | 0 |
| MI | 8/29/2007 | 2 | 0 | 0 | 0 | 0.5 | 0 | 0 | 0 |
| MI | 8/29/2007 | 2 | 0 | 0 | 0 | 0 | 0 | 0 | 0 |
| MI | 8/29/2007 | 3 | 1 | 0.5 | 0 | 0 | 0 | 0 | 0 |
| MI | 8/29/2007 | 2 | 0.5 | 0 | 0 | 0 | 0 | 0 | 0 |
| MI | 8/29/2007 | 3 | 0 | 1 | 0 | 0 | 0 | 0 | 0 |
| MI | 8/29/2007 | 3 | 0 | 1 | 0 | 0 | 0 | 0 | 0 |
| MI | 8/29/2007 | 3 | 0 | 0 | 0 | 0.5 | 0 | 0 | 0 |
| STK | 9/12/2007 | 1 | 0 | 0 | 0 | 0 | 0 | 0 | 0 |
| STK | 9/12/2007 | 1 | 0 | 0 | 0 | 0 | 0 | 0 | 0 |
| STK | 9/12/2007 | 1 | 0 | 0 | 0 | 1 | 0 | 0 | 0 |
| STK | 9/12/2007 | 3 | 0 | 0 | 0 | 0 | 2 | 0 | 1 |
| STK | 9/12/2007 | 3 | 0 | 0 | 0 | 0.5 | 1 | 0 | 0 |
| STK | 9/12/2007 | 2 | 0 | 0 | 0 | 0.5 | 2 | 0 | 0 |
| STK | 9/12/2007 | 2 | 0 | 0 | 0 | 1 | 2 | 0 | 0 |
| STK | 9/12/2007 | 2 | 0 | 0 | 0 | 0 | 2 | 0 | 0 |
| STK | 9/12/2007 | 2 | 0 | 1 | 0 | 0.5 | 2 | 0 | 0 |
| STK | 9/12/2007 | 3 | 0.5 | 0 | 0 | 0 | 1 | 0 | 0 |
| STK | 9/12/2007 | 3 | 0 | 0 | 0 | 0.5 | 2 | 0 | 0 |
| STK | 9/12/2007 | 3 | 0 | 0 | 0 | 0 | 2.5 | 0 | 0 |
| STK | 9/12/2007 | 2 | 0 | 0 | 0 | 0 | 0 | 0 | 0 |
| STK | 9/12/2007 | 3 | 0 | 0 | 0 | 0 | 2 | 0 | 0 |
| STK | 9/12/2007 | 1 | 0 | 0 | 0 | 0 | 0 | 0 | 0 |
| STK | 9/12/2007 | 2 | 0 | 0 | 0 | 0 | 1 | 0 | 0 |
| STK | 9/12/2007 | 3 | 0.5 | 0 | 0 | 1 | 3 | 0 | 0.5 |
| STK | 9/12/2007 | 2 | 1 | 0 | 0 | 1 | 2 | 0 | 0 |
| STK | 9/12/2007 | 1 | 0.5 | 0 | 0 | 0 | 1 | 0 | 0 |
| STK | 9/12/2007 | 2 | 0 | 0 | 0 | 0 | 1 | 0 | 0 |
| SI | 9/11/2007 | 0 | 0.5 | 0 | 0 | 0 | 0 | 0 | 0 |
| SI | 9/11/2007 | 0 | 0 | 0 | 0 | 0 | 0 | 0 | 0 |
| SI | 9/11/2007 | 0 | 0 | 0 | 0 | 0.5 | 0 | 0 | 0 |
| SI | 9/11/2007 | 2 | 0 | 2 | 0 | 0 | 2 | 0 | 0 |
| SI | 9/11/2007 | 0 | 0 | 0 | 0 | 0 | 0 | 0 | 0 |
| SI | 9/11/2007 | 0 | 0 | 0 | 0 | 1 | 0 | 0 | 0 |
| SI | 9/11/2007 | 1 | 0 | 1 | 0 | 0 | 0 | 0 | 0 |
| SI | 9/11/2007 | 1 | 0 | 0 | 0 | 0 | 0 | 0 | 0 |
| SI | 9/11/2007 | 0 | 0 | 0 | 0 | 0 | 0 | 0 | 0 |
| SI | 9/11/2007 | 0 | 0 | 0 | 0 | 0 | 0 | 0 | 0 |
| SI | 9/11/2007 | 0 | 0 | 0 | 0 | 2 | 0 | 0 | 0 |
| SI | 9/11/2007 | 0 | 0 | 0 | 0 | 0 | 0 | 0 | 0 |
| SI | 9/11/2007 | 1 | 0 | 0 | 0 | 0 | 0 | 0 | 0 |
| BI | 9/11/2007 | 2 | 1 | 1 | 0 | 1 | 0 | 0 | 0 |
| BI | 9/11/2007 | 0 | 0 | 0 | 0 | 0 | 0 | 0 | 0 |
| BI | 9/11/2007 | 2 | 0 | 1 | 0 | 0 | 0 | 0 | 0 |
| BI | 9/11/2007 | 1 | 0 | 3 | 0 | 0 | 0 | 0 | 2 |
| BI | 9/11/2007 | 1 | 0 | 0 | 0 | 0 | 0 | 0 | 0 |
| BI | 9/11/2007 | 3 | 0 | 3 | 0 | 0 | 0 | 0 | 0 |
| BI | 9/11/2007 | 0 | 0 | 1 | 0 | 1 | 0 | 0 | 1 |
| BI | 9/11/2007 | 0 | 0 | 0 | 0 | 0 | 0 | 0 | 0 |
| BI | 9/11/2007 | 2 | 0 | 1 | 0 | 0 | 0 | 0 | 2 |
| BI | 9/11/2007 | 1 | 0 | 0 | 0 | 1 | 0 | 0 | 1 |
| BI | 9/11/2007 | 1 | 0 | 0 | 0 | 1 | 0 | 0 | 0 |
| BI | 9/11/2007 | 3 | 0 | 0 | 0 | 0 | 0 | 0 | 0 |
| BI | 9/11/2007 | 3 | 0 | 2 | 0 | 0 | 0 | 0 | 0 |
| BI | 9/11/2007 | 1 | 0 | 1 | 0 | 0 | 0 | 0 | 0 |
| BI | 9/11/2007 | 3 | 1 | 0 | 0 | 1 | 0 | 0 | 2 |
| BI | 9/11/2007 | 1 | 0 | 1 | 0 | 0 | 0 | 0 | 2 |
| BI | 9/11/2007 | 2 | 0 | 2 | 0 | 0 | 0 | 0 | 0 |

Table S3. Proximate analysis (%) for protein by dry weight, lipid by dry weight, and moisture measured for Threadfin shad collected at Sherman Island (SI), Mildred Island (MI), Brannan Island (BI), and Stockton (STK).

| **Site** | **Wet weight** | | | **Dry Weight** | |
| --- | --- | --- | --- | --- | --- |
|  | **Moisture** | **Protein** | **Lipid** | **Protein** | **Lipid** |
| SI | 76.86 | 15.22 | 1.71 | 65.79 | 7.37 |
| SI | 77.98 | 14.83 | 1.84 | 67.35 | 8.37 |
| SI | 75.98 | 14.88 | 3.18 | 61.96 | 13.25 |
| SI | 75.81 | 14.78 | 2.33 | 61.10 | 9.62 |
| MI | 79.96 | 14.94 | 0.43 | 74.53 | 2.15 |
| SI | 73.16 | 15.46 | 3.09 | 57.60 | 11.51 |
| SI | 73.12 | 15.84 | 2.33 | 58.92 | 8.66 |
| SI | 74.11 | 15.75 | 2.52 | 60.82 | 9.73 |
| BI | 76.49 | 16.10 | 1.27 | 68.50 | 5.41 |
| BI | 70.50 | 15.61 | 8.11 | 52.90 | 27.50 |
| BI | 74.93 | 15.85 | 1.72 | 63.22 | 6.86 |
| BI | 76.17 | 15.85 | 2.17 | 66.52 | 9.11 |
| STK | 79.19 | 14.35 | 0.90 | 68.97 | 4.34 |
| STK | 78.95 | 14.59 | 0.96 | 69.33 | 4.55 |
| STK | 78.23 | 14.70 | 1.21 | 67.53 | 5.54 |
| STK | 77.68 | 15.39 | 1.16 | 68.94 | 5.22 |

Fig. S1. Histopathology of Threadfin shad, *Dorosoma petenense*, from the bloom sites, showing a) severe glycogen depletion and lipidosis at 400x and b) fatty vacuolar degeneration (arrows) at 400x.

Fig. S2. *In situ* hybridization (ISH) and H&E staining of Threadfin shad (TFS), *Dorosoma petenense*, from Bannon Island in the upper San Francisco Estuary. A) H&E staining of the intestine at 100x and b) by ISH showing ingested *Microcystis* cells (arrows) staining blue in gut contents and in between intestinal wall and lining of TFS 100x.

Fig. S3. *In situ* hybridization (ISH) and H&E staining in the stomach of Threadfin shad (TFS), *Dorosoma petenense*, from Brannon Island in the upper San Francisco Estuary. A) H&E staining of the stomach of TFS. B) Blue precipitates indicate ingested *Microcystis* cells (arrows) by ISH in the stomach contents of TFS at 100x.
